# Home Range, Movement, and Distribution Patterns of the Threatened Dragonfly *Sympetrum depressiusculum* (Odonata: Libellulidae): A Thousand Times Greater Territory to Protect?

**DOI:** 10.1371/journal.pone.0100408

**Published:** 2014-07-09

**Authors:** Aleš Dolný, Filip Harabiš, Hana Mižičová

**Affiliations:** 1 Department of Biology and Ecology/Institute of Environmental Technologies, Faculty of Science, University of Ostrava, Ostrava, Czech Republic; 2 Department of Ecology, Faculty of Environmental Sciences, Czech University of Life Sciences Prague, Prague, Czech Republic; Université de Sherbrooke, Canada

## Abstract

Dragonflies are good indicators of environmental health and biodiversity. Most studies addressing dragonfly ecology have focused on the importance of aquatic habitats, while the value of surrounding terrestrial habitats has often been overlooked. However, species associated with temporary aquatic habitats must persist in terrestrial environments for long periods. Little is known about the importance of terrestrial habitat patches for dragonflies, or about other factors that initiate or influence dispersal behaviour. The aim of this study was to reveal the relationship between population dynamics of the threatened dragonfly species *Sympetrum depressiusculum* at its natal site and its dispersal behaviour or routine movements within its terrestrial home range. We used a mark–release–recapture method (marking 2,881 adults) and exuviae collection with the Jolly–Seber model and generalized linear models to analyse seasonal and spatial patterns of routine movement in a heterogeneous Central European landscape. Our results show that utilisation of terrestrial habitat patches by adult dragonflies is not random and may be relatively long term (approximately 3 mo). Adult dragonflies were present only in areas with dense vegetation that provided sufficient resources; the insects were absent from active agricultural patches (*p* = 0.019). These findings demonstrate that even a species tightly linked to its natal site utilises an area that is several orders of magnitude larger than the natal site. Therefore, negative trends in the occurrence of various dragonfly species may be associated not only with disturbances to their aquatic habitats, but also with changes in the surrounding terrestrial landscape.

## Introduction

The population ecology of dragonflies is among the best known of all freshwater insects. Dragonfly larvae live in a variety of freshwater habitats, including lakes, bogs, seepages, rivers, and springs. It is not surprising, therefore, that the majority of effective conservation strategies for threatened dragonfly species are designed to protect freshwater habitats [1]. The question remains, however, as to whether protection of aquatic habitats is sufficient or whether it is necessary to also include the terrestrial environments surrounding natal habitats. If terrestrial areas should also be protected, how large should these protected zones be?

Not only larvae, but also adults can be very sensitive to changes in their environment [2]–[5]. Adult odonates can reflect minor anthropogenic disturbances that occur in terrestrial ecosystems (e.g. logging in forest areas) even when there is no direct disturbance of aquatic habitat [4]. Dragonfly adults seek specific microhabitats in terrestrial environments for foraging, resting, and reproduction. The availability of particular habitat patches can be essential for the long-term survival of many insect species [6]. As a result of increasing urbanization and habitat fragmentation, numerous studies highlighting the effects of habitat and landscape quality have been conducted, focusing especially on the influence of landscape structure and heterogeneity on diversity, population stability, movement, and risk of local extinction of a number of insect taxa [6]–[8].

In aquatic insects, terrestrial habitats are often used for diffuse movements of individuals from their natal habitat or home district into new territory (i.e. dispersal) [9]. The dispersal ability of most insect groups is very limited; comprehensive understanding of dispersal ability is important for effective management of endangered species [10]. The dispersal ability of dragonflies reflects species habitat specificity [11], but is also influenced by local environmental conditions, as is true for other major groups of flying insects [12]. It has been pointed out that data on dispersal behaviour acquired through capture–mark–recapture (CMR) studies may be of limited value because these studies are distinctly biased towards examination of routine movements [13]. Especially in conservation biology, a great deal of attention is devoted to the distances that organisms can travel and to connectivity between habitats, but very little is known about the mechanisms that precede a dispersal decision and perhaps even less attention is devoted to the habitat-selection process [14].

The value of terrestrial environments for dragonflies is substantially underappreciated. Although several studies have shown the significant influence of dragonflies on terrestrial ecosystems [15] or the effects of various factors in terrestrial environments on dragonflies (e.g. [16]), very little is known about the effect of individual characteristics of terrestrial environments on the distribution of odonates. The majority of studies addressing factors that affect dragonfly distribution have focused on surface waters and their immediate surroundings [17], [18]. Macrophytic vegetation surrounding water bodies is the most commonly cited ‘terrestrial’ factor that can positively or negatively affect dragonfly distribution according to its presence or absence [19]. While vegetation can serve as an important shelter and foraging place, it may also constitute a barrier to dispersal of flying adults [9], [20]. A number of studies have examined the influence of biotic and abiotic patterns on dragonfly diversity [18], but the importance of the surrounding terrestrial environment (the prevailing land uses outside of riparian zones, such as woodlands, shrubs, grasslands, or arable fields) is often overlooked or cannot be further conclusively interpreted (see [21]).

The importance of certain habitat patches in the landscape matrix may be particularly relevant for species occurring in temporary habitats, such as *Sympetrum depressiusculum*, a threatened dragonfly species whose populations in Central and Western Europe are greatly scattered and spatially isolated. Although *S. depressiusculum* is regarded as sedentary, the imagoes spend most of their lives (the majority of the pre-reproductive period) outside of water bodies [22]. Therefore, it can be assumed that the composition, distribution, and heterogeneity of habitat patches in areas surrounding aquatic habitats will affect the population dynamics and habitat selection of this species. In other words, there should be a preference for one or more types of terrestrial environments within the landscape matrix.

In the present study we aimed to gain better understanding of how important are the terrestrial habitats for dragonflies. Our main question was whether the composition, distribution and the heterogeneity of habitat patches in the surroundings of natal aquatic habitat can affect the population dynamics and the habitat selection of adult dragonflies. We also investigated the preference for a certain types of terrestrial environment within landscape matrix conditions for adults.

## Methods

### Ethics statement

No specific permits were required for fieldwork as the sampled localities are not protected. The natal pond and its surroundings are owned by the Czech Fishing Union, and the data were collected with the approval of one of the members (Milan Konvička). No specific permissions were required to collect dragonflies, because the collected species (*Sympetrum depressiusculum*) is endangered but not protected in the Czech Republic. In Europe, *S. depressiusculum* has been assessed by the IUCN as vulnerable.

### Study site

**Figure 1:**
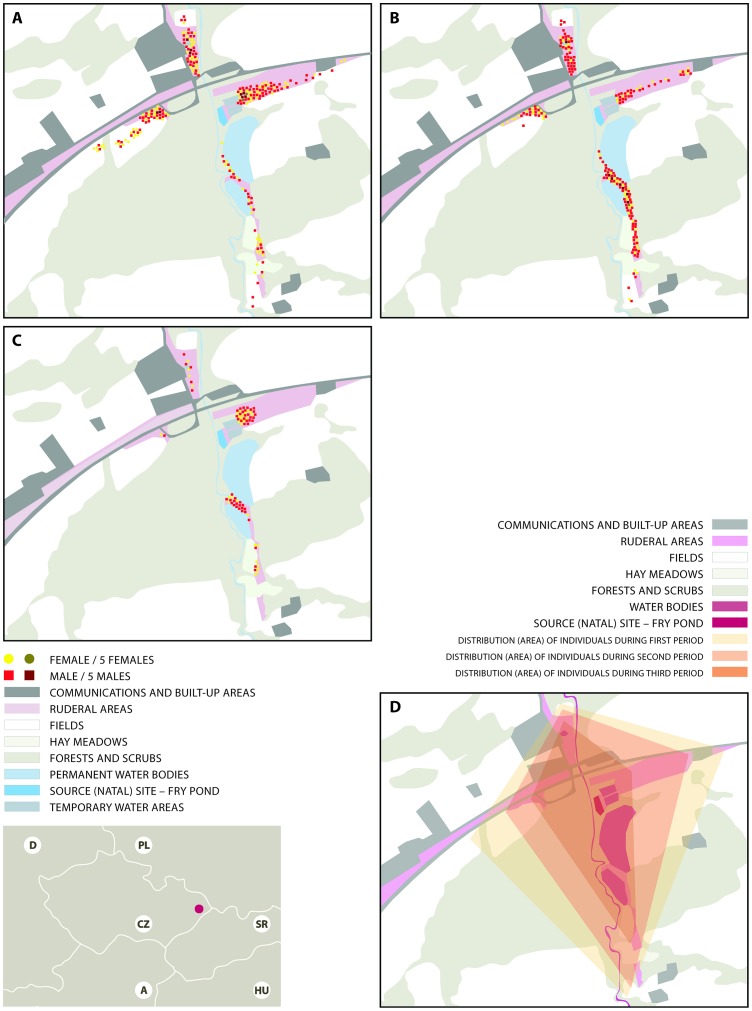
Map of the study area showing the four research transects, locations of individual records of adults, and habitat preferences of adults. A) 7 July–8 August 2012; B) 9 August–6 September 2012; C) 7 September–10 October 2012; D) home-range extent divided into three time stages, see previous description – A, B, C. Adults were present only on heavily vegetated surfaces and were absent from areas in agricultural production patches after the harvest.

The research was conducted from the beginning of July to the end of September 2012 in a 3-km^2^ area in the north-eastern Czech Republic (Fig. 1). The study area is located in the foothills of the Beskydy Mountains (approximately 300 m a.s.l.) and is composed primarily of agricultural landscape, with remnants of floodplain forest in the River Sedlnice catchment (Fig. 1). Larval development of *S. depressiusculum* was observed at a single natal site, a 3,000-m^2^ pond (49°38′5.51″N, 18°6′4.14″E). This pond has been used for >20 years to rear the phytophagous cyprinid fish *Chondrostoma nasus*, which requires specific management practices, including periodic draining of the pond in late summer to promote growth of aquatic vegetation. The terrestrial landscape surrounding the natal pond is heterogeneous and includes agricultural areas (cereal and root crops), grasslands (hay meadows, abandoned grasslands and fields, stream banks), and forest (natural softwood floodplain forests, oak–hornbeam woodlands).

### Study species

*Sympetrum depressiusculum* (Sélys, 1841) is threatened throughout Europe and occurs in scattered populations from Siberia and Japan to Western Europe. This species prefers small, temporary aquatic habitats, especially those in alluvial areas of rivers and lakes and in waterlogged meadows, but it also occurs in anthropogenic habitats including winter-dry fish ponds and rice fields [23], [24]. The emergence period in central Europe starts at the beginning of July and continues through mid-August. In good weather conditions, the flight period ends in mid-October. Although *S. depressiusculum* is considered endangered, it can be locally abundant; several records describe massive occurrences of individuals outside of water bodies during the pre-reproductive period [24]. *S. depressiusculum* is one of four threatened dragonfly species in Europe that are not confined to the Mediterranean [1] and that have declining populations and restricted distribution in Western Europe [25].

### Demography of natal population

Studies increasingly reveal that information about population size and other population parameters for individual dragonfly species, and biodiversity assessments conducted using adult surveys, can be very biased (e.g. see [26]). We therefore complemented our estimates based on CMR methods with data derived from exuviae sampling, which was advantageous in that these data were not weather-dependent and they provided an accurate determination of sex ratios for comparison of numbers of emerged individuals and adults returning to the natal site. Between 29 June and 6 September 2012 (the entire emergence period), exuviae were collected every 3 d from littoral vegetation along 12 randomly selected transects, which comprised approximately 5% of the shoreline. Each transect was 1 m wide×2 m long. Exuviae sampling provided a more precise measure of population size and enabled us to determine population parameters including sex ratio or emergence phenology. Exuviae were preserved in labelled glass containers, identified to species level (additional *Sympetrum* species occurred at the sampling site) and sex, and counted; species determinations followed Gerken and Sternberg [27].

The CMR study was conducted between 7 July and 26 September 2012. During the flight season for adult *S. depressiusculum*, 39 individual walks were made along the embankment through 5-m wide transects. Each captured individual was marked on the wings using a unique code and permanent marker. Individuals recaptured during the same day of marking were not counted. Marking was carried out at regular time intervals; the length of individual walks around the perimeter of the pond was equal to avoid uneven distribution of samples in time (season) and space.

### Habitat use and dispersal

We established four longitudinal transects (extending four directions from the natal site) through the landscape mosaic. Each transect was 1500 m long, started approximately 100 m from the breeding site, and was virtually divided into 10-m subsections. The original length of transects was not fixed, because the goal was to capture individuals with maximum dispersal ability. Each subsection was searched for unmarked and marked individuals of both sexes. Captured individuals were marked and later released into the middle of the subsection within which they were captured. Each 10-m subsection was assigned to a particular habitat type. We distinguished the following 5 habitat types, which were clearly identifiable based on vegetation and land use: a) permanent grasslands, b) agricultural production fields, c) abandoned fields, d) small-scale ruderal areas (ruderals), and e) wetland areas with riparian vegetation. Habitat use was evaluated according to the number of individuals marked in patches of each habitat type.

The proportions of individual habitat types differed among transects because of the landscape's spatial arrangement, but were balanced when considering the cumulative transects. The examined flight period (88 d) was divided into three equal parts: 7 July–8 August (29 d), 9 August–6 September (29 d), and 7 September–10 October (30 d).

### Data analyses

#### Population size and dynamics

We analysed mark–recapture data to evaluate the demographics of the *S. depressiusculum* population using the constrained linear model in the MARK program, version 6.1 [28], [29]. In MARK, we applied the Jolly–Seber method and POPAN parameterization for open population parameter estimates [30]. This model estimates three primary parameters: *φ_i_* (residence), which combines the probability of an animal surviving between occasions *i* and *i*+1, and thus essentially combines mortality and emigration rates; *p_i_* (catchability); and *pent_i_*, which is the probability of entering the population (a combination of birth and immigration). The primary parameters can be independent of both sex and time, can differ between sexes (*g*), or can be dependent on time in a factorial (*t*), linear (*t_lin_*), or polynomial (*t^2^_lin+lin_*) manner. Sex–time interactions can be either multiplicative (*g*×*t*) or additive (*g+t*). Complex polynomial models were calculated and subsequently simplified. Model selection was based on an information-theory approach, using corrected Akaike information criterion (AICc). The model with the lowest AICc value was chosen as the final model. Models that had ΔAICc ≤2 in comparison to the best model were considered as equivalent [31]. Based on these parameters, several derived parameters can be estimated: *λ_i_* is the population growth rate between sample times *i* and (*i+1*), with population size *N_i_* and total number of individuals *N_tot_* at time *i*. Average catchability (*p*′) and residence (*φ*′) are simple arithmetic means from POPAN daily values. Longevity is calculated as −ln(*φ*′)^−1^ [32].

All *S. depressiusculum* adults (both sexes) captured more than once were used for regression-based models to fit cumulative probability of movement (*I*) against movement distance (*D*) for [33]:
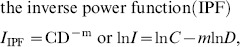
where parameters *C* and *m* are estimated by fitting the logarithms of cumulative fractions of individuals moving to certain distances against the logarithms of those distances, and
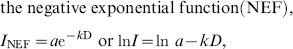
where *a* and *k* are parameters estimated by fitting the logarithms of cumulative fractions of individuals moving to certain distances against those distances.

#### Habitat use and dispersal

The preferences for certain habitat patch types and the spatial and temporal variation of adult males and females were analysed using generalized linear models (GLM). A model with Gaussian distribution errors was used to analyse the effects of sex, period, and transect (explanatory variables) on distance from the natal site where the individual had been marked (response variable). In a second model with quasi-Poisson errors, we analysed the relationship between the abundance of individuals (response variable) and sex, habitat, and period as explanatory variables.

To select the best-fit models, we made an assessment with the AICc using the glmulti function of glmulti 1.0.7. [34]. Models with ΔAICc ≤2 in comparison with the best model were again considered as equivalent [31]. The best models were verified using standard statistical diagnostics: residuals versus fitted values, distribution of standardized residuals, homogeneity of residual variance, and Cooke distances [35]. Statistical significance was established using α = 0.05. Post-hoc comparisons of pairwise differences between the mean abundance of individuals in different habitat types and periods were made using Tukey contrasts for multiple comparisons of means [36]. All analyses were conducted in R 2.13. [37].

## Results

A total of 1991 males and 268 females were marked, of which 171 males (8.59%) and 2 females (0.75%) were recaptured at the natal site (Table 1). We collected 3863 exuviae (1899 males and 1964 females) at the natal site. Although emergence began on 29 June and ended on 6 September, most of the population (>90%) emerged during the first 2 wk of the emergence period. The male-to-female sex ratio was 0.97 (based on exuviae collection); 622 individuals (413 males and 209 females) were marked on transects, but only 38 (33 males and 5 females) were recaptured on transects.

**Table 1:** Numbers of individual *Sympetrum depressiusculum* marked and recaptured at the natal site.

Marking days	Marked (♂/♀)	Recaptured (♂/♀)	Capture events (♂/♀)
39	1991/268	148/1	2153/269

### Demography of the natal population

The population size estimated using the Jolly–Seber method was 57,949 individuals after model averaging (Table 2), while we obtained an estimate of 77,279 individuals by converting the mean number of exuviae (322) per m^2^. Both estimates indicated a female-biased sex ratio, but the more reliable estimate obtained from exuviae collection pointed to a very narrow female-bias (0.97).

**Table 2:** Results of the Jolly–Seber analysis (POPAN module in MARK): selected models, numbers of model parameters (K), estimates of average longevity (*Long.*) [days], and total estimated population size (*N*) for male and female *Sympetrum depressiusculum*.

Model*	*AIC*	*ΔAIC*	K	*Long.* (♂/♀)	*N*♂ (± SE)	*N*♀ (± SE)
*φ(.) p(g+t) pent(g+t^2^_lin+lin_) N(.)*	2456.75	0.00	48	2.38/3.04	26267±8168	32734±7986
*φ(t_lin_) p(g+t) pent(g+t^2^_lin+lin_) N(.)*	2458.50	1.75	49	2.38/3.00	27301±6685	33904±8296
Model averaging					27061±7115	30888±13795

* Models were selected according to the Akaike information criterion (AIC). The model with the lowest AIC value and the model with ΔAICc ≤2.00 are shown.

**Figure 2:**
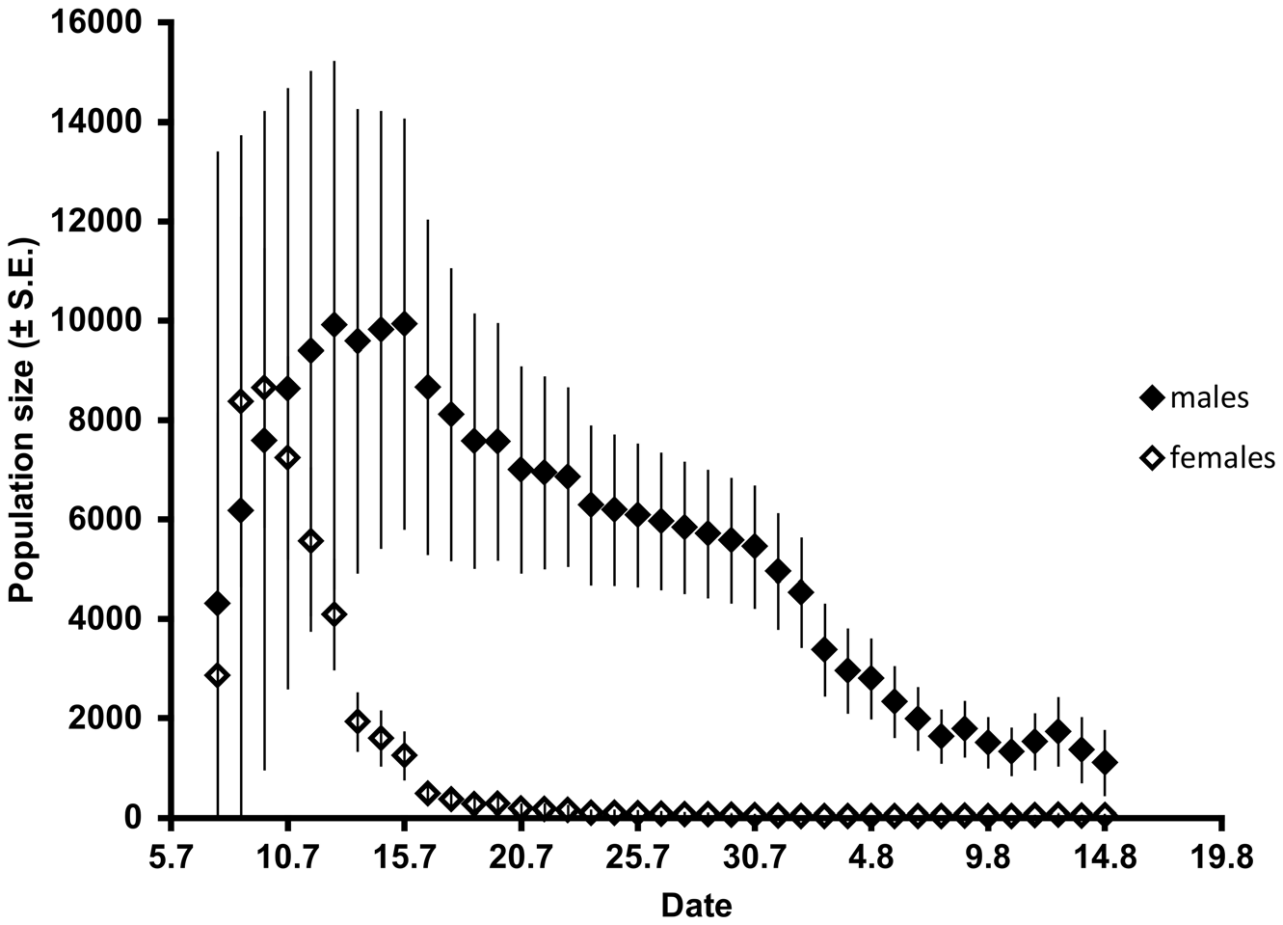
Daily estimates of *Sympetrum depressiusculum* population size at the natal site.

The best-fit models indicated that residence (*φ*) was linearly time-dependent or was constant and independent of sex. Catchability (*p*) was both time- and sex-dependent, because females remained at the site only briefly after emergence while males occasionally returned to the site (Fig. 2). Recruitment (*pent*) showed a polynomial response and was sex-dependent. The average estimated daily survival was 2.38 d for males and 3.04 d for females, but the longest-recorded living individual was a male that was recaptured 59 d after it had been marked.

### Habitat use and dispersal

**Figure 3:**
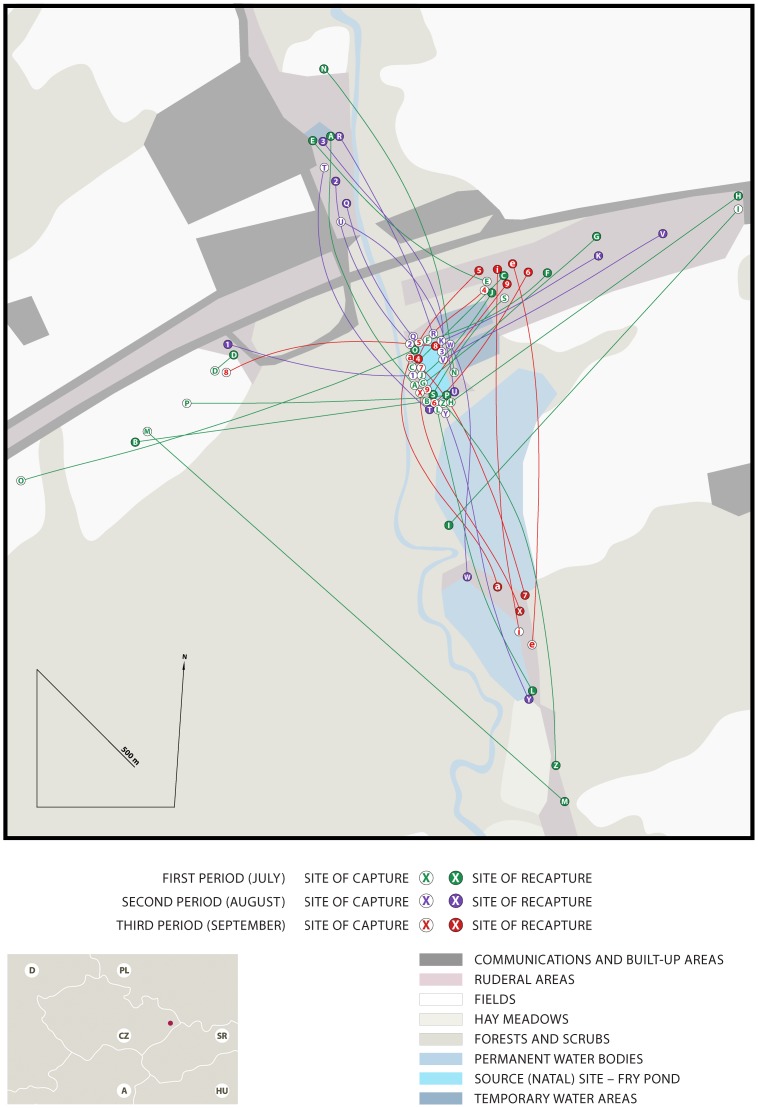
Schematic representation of all recorded moves between natal site and terrestrial patches. There are only five females (E, H, J, T and 8), all others are males.

**Figure 4:**
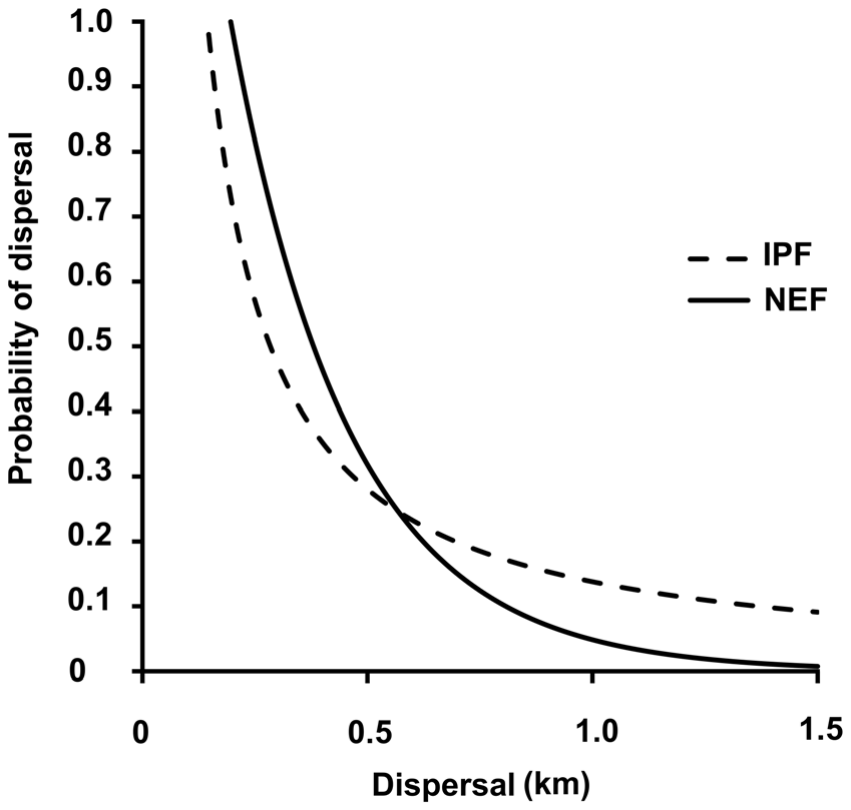
Probability of individual adult *Sympetrum depressiusculum* dispersing a given distance, estimated from regression-based models (inverse power function, IPF; negative exponential function, NEF).

The dispersal, movement, and other activities of adult *Sympetrum depressiusculum* within the entire non-breeding area were recorded throughout the flight period (11 July to 6 October 2012). The maximum recorded distance from the natal site was 1196 m, while the maximum distance travelled between segments was 1128 m, both of which distances were recorded for males (Table 3). The average distance of females from the natal site was significantly greater than that of males (481 and 446 m, respectively). Females were captured throughout the season in transects, unlike at the natal site. We recorded 33 movements of males, and only 5 movements by females, on transects (Fig. 3). The majority of dispersal events were related to movements between the natal site and the terrestrial environment; 19 movements from the natal site to the terrestrial environment were recorded and 7 movements were recorded in the opposite direction. Only three dispersal events between terrestrial patches were recorded. The remaining overflights were probably exploratory movements aimed at colonization of new aquatic habitats. Specifically, these occurred at two intensively managed ponds on the south transect, approximately 650 m from the natal site (Fig. 3). Based on the NEF model (which had better predictive power than the IPF model), fewer than 5% of individuals flew >1 km, but more than 30% of individuals dispersed >0.5 km. (Table 4; Fig. 4).

**Table 3:** Numbers of marked and recaptured *Sympetrum depressiusculum* on each transect, and mean and maximum distances travelled in the landscape.

Transect	Marking days	Marked (♂/♀)	Recaptured (♂/♀)	Mean distance (♂/♀)	Max. distance (♂/♀)
N	13	94/33	8/3	448/442	644/663
E	13	122/62	12/0	278/338	623/845
S	13	142/62	8/1	588/645	1196/1170
W	13	55/52	5/1	448/480	842/788
∑	26	413/209	33/5	446/481	1196/1170

**Table 4:** Probability of individual adult *Sympetrum depressiusculum* dispersing a given distance, estimated from regression-based models (inverse power function, IPF; negative exponential function, NEF) and observed data.

Model	Fitted function	R2	0.5 km	1.0 km
IPF	ln*I* = −1.98 −1.03ln*D*	0.61	0.2805	0.1379
NEF	ln*I* = −0.74 −3.77*D*	0.94	0.3185	0.0484
Observed			0.3421	0.0263

R^2^ is the coefficient of determination.

Movements directed from the natal site towards terrestrial environments were recorded throughout most of the adult flight period. In contrast, ‘return’ flights (i.e. movements towards the natal site) were recorded only in the second half of August and in September. The longest time interval between detection of an individual at a terrestrial habitat and its recapture at the natal site was 57 d. The appearance of marked individuals at other permanent water bodies occurred only during the last third of the flight period.

**Figure 5:**
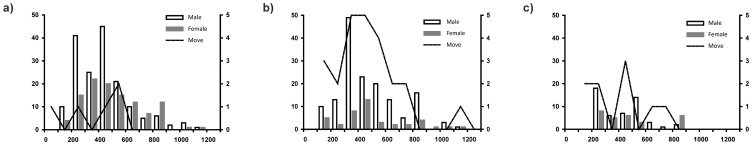
Relationship between number of *Sympetrum depressiusculum* (both sexes) and distance from the natal site, and graphical representation of recorded movements of marked adults (“Move”) according to distance. A) 7 July–8 August 2012; B) 9 August–6 September 2012; C) 7 September–10 October 2012.

Also, the distance from the natal site and distribution of individuals changed over time (Fig. 5). The most parsimonious model {*distance ∼ 1 + sex + transect + period*} (AICc = 8069.74; AICc weights = 0.856) revealed significant effects of period and sex on the distribution of individuals, while the distribution of individuals assessed as distance from the natal site differed significantly between the transects (Tables 4, 5). The greatest change (decline) in the distribution of individuals occurred between the first and second periods (t = −2.940; *P* = 0.009), whereas the change in the distribution of individuals between the second and third periods was not significant (*t* = 2.096; *P* = 0.087).

**Table 5:** Significance of selected factors to the distribution of *S. depressiusculum* individuals.

Variable	Df	X^2^	F	*P*
Sex	1	0.71	6.50	0.017
Transect	4	61.12	140.78	<0.001
Period	2	1.78	8.18	<0.001

Adults showed some degree of preference for patches of certain habitat types. Different distributions of individuals among transects was likely a result of the different representation of individual habitat types within the landscape matrix. The most parsimonious model {*abundance ∼ 1 + sex + habitat + period*} (AICc = 901.35; AICc weight = 0.525) revealed significant differences in preference for certain types of habitat patches (Table 6). Individuals of both sexes preferred small ruderal patches (t = 3.017; *P* = 0.019) or abandoned fields (t = 2.856; *P* = 0.031) over meadows. Agricultural fields were used only prior to harvesting (Fig. 1A). After the harvest, *S. depressiusculum* was almost entirely absent from agricultural fields and from regularly cut hay meadows. There was a significant decrease (t = −2.611; *P* = 0.024) in the number of individuals observed on transects between the second and third periods, which may have occurred because the flight period ended, or because the majority of individuals had returned to the natal site or relocated to other water bodies (Fig. 1B,C) for reproduction.

**Table 6:** Significance of selected factors on the abundance of *Sympetrum depressiusculum* within different habitat patches.

Variable	Df	Deviance	*P*
Sex	1	68.27	0.017
Habitat	4	259.34	<0.001
Period	2	148.06	0.002

## Discussion

Our data support the concept of using dragonflies as indicators of freshwater habitat quality and changes in habitat quality [4], [38], [39]. In addition, our findings suggest that the structure of the surrounding landscapes (i.e. availability of certain habitat patches) may be crucial for adult dragonflies and that heterogeneity of terrestrial habitats should thus be taken into account in conservation management. Positive relationships between vegetation-based landscape/habitat heterogeneity and species richness of insects is well documented on regional scales, and also on micro- and meso-scales [40], [7]. For instance, landscape heterogeneity (e.g. within-habitat heterogeneity of vegetation) is a principal factor determining butterfly species richness [8], [41] and habitat heterogeneity is positively associated with stability of butterfly populations and lower risk of extinction [6]. Similarly, land use and the structure of vegetation adjacent to aquatic habitats (especially important as nocturnal roosts) have a dominant influence on odonate diversity [42] and abundance of adults [42], [43], and on fine-scale movement behaviours of damselflies [44].

Our results suggest that the structure of habitat patches outside of freshwater habitats can be important for major life events in *S. depressiusculum*, especially juvenile development and routine movements of imagoes at sexual maturity. The use of terrestrial habitats in adults of this species was long term – even exceeding 3 months, which is at least as long as the period of the larval development. These findings are consistent with previous studies suggesting that dragonflies play a significantly role in terrestrial food chains [45], [46]. Nevertheless, little attention is given to the use of terrestrial habitats by adult dragonflies, and the role of these habitats in the life cycle. This may be due to a presumption that the dramatic decline in distribution and abundance of many dragonfly species in temperate climates since the second half of the 20th century is mainly a result of destruction of aquatic habitats [47], and that protection of these habitats should thus be the primary focus. Our study reveals that imagoes make limited use of aquatic habitats and their immediate surroundings, and that the area used over the long term (the non-breeding home range) may extend approximately 0.5–1.0 km from the natal site (thus the area utilized by this in our study was up to 1000-fold greater than the area of the natal site).

### Demography

Dispersal patterns and behaviours of *S. depressiusculum* clearly differed by sex and changed during the season. It is generally assumed that, similar to other freshwater invertebrates, the dispersal behaviour of dragonflies is density-dependent (e.g. [48], reviewed by [49]) and that differences in dispersal behaviour between the sexes can be explained by the lek mating-system hypothesis [50]. In most dragonfly species, territoriality manifests in the behaviours of males attempting to guard an appropriate territory [9]. Males with established territories have a lower tendency to disperse, but their density is limited by the number of territories available [51]. However, several dragonfly species, including *S. depressiusculum*, exhibit a different strategy wherein high densities of conspecific males tend to stay at a breeding site and distribute around the aquatic habitat. A high density of males at a breeding site influences the routine movements of females, who leave the natal site immediately after emergence and return only very briefly for reproduction [52].

Although females moved away from the water body and the recapture rates for females were relatively low, none of our data indicated that females passed the distance that marks a departure from the natal site. Rather, it can be assumed that female behaviour was a reaction to a lack of resources and/or harassment by males [53]–[56]. The majority of the *S. depressiusculum* population emerges during the first few days of the flight period [22], meaning that the population is not progressively augmented by newly emerging individuals. This may lead to reduced population density and a gradual decrease in size of the territory towards the end of the season. We did not record any individuals (male or female) moving beyond the critical distance, which suggests that the lower number of females marked on transects was related to the female's rather more hidden lifestyle. This idea is not new and has been suggested previously in several studies [57].

### Habitat use

As winged insects, dragonflies have a relatively large radius of action, and the aquatic habitat and immediate surroundings represent a small fraction of the area utilized by these species. Terrestrial habitats, while not suitable for larval development, provide forage and shelter [9] and so are essential to survival. Our study indicates that even terrestrial habitat patches within an agricultural matrix provide a mosaic of preferred and disfavoured habitats.

Adult *S. depressiusculum* clearly avoided agricultural areas that were in production (farm fields and especially hay meadows). This could be explained by the type and especially the structure (height and density) of vegetation; vegetation structure may to be more important than plant species composition, as has been observed for butterflies [42], [43]. A number of studies have highlighted the significant effects of macrophytes as keystone structures influencing dragonfly diversity [9], [18], [42]. Our findings support the generally applicable hypothesis that the potential for agricultural landscapes to provide sufficient shelter or forage is significantly reduced by on-going disturbances, especially harvesting [58]. In this study, dragonflies preferred ruderal patches and abandoned fields. A field does not need to be abandoned for an extended period of time to provide habitat; the important factor is the absence of continual disturbance during the adult flight period.

In studies based on mark and recapture, it is difficult to exclude the possibility that part of the population (the dispersers) leaves the site and that the focus is biased towards residents [59]. In many cases, it is difficult to distinguish between a dispersal event and other types of behaviours that represent routine movements within home-range territories [13]. Thus, it may be difficult to estimate the distance that can be routinely travelled by a given species. Our field observations suggest that our presumption of high natal philopatry in *S. depressiusculum* is valid [22]. This idea is supported by several findings: 1) the distances of individuals marked at the points farthest from the natal site were very similar (approximately 1 km) in all directions and on all transects, and did not cross the critical distance; 2) the external borders of the home range were diminished during the flight season; and 3) the numbers of emerged individuals and adults returning to the natal site were comparable, based on exuviae collection and capture–mark–recapture.

Very little is known about the spatial orientation of dragonflies. It is thought that the ability to perceive polarized light plays an important role in spatial orientation over longer distances and that the overall character of (aquatic) vegetation is important at shorter distances [60], [61]. The effects of other landscape elements on spatial orientation in insects are poorly understood. Based on our field observations, we assume that even less ideal aquatic habitats, such as temporary pools and fish ponds, have an important function and can be used as stepping stones for subsequent dispersal. In addition, the structure of terrestrial habitats appears to have a considerable effect on dragonflies and other water-breeding invertebrates, and thus should be considered and included in conservation planning.
